# Hyaluronic acid-*b*-polylactic acid polymersomes facilitate CD44-mediated delivery of doxorubicin to glioblastoma *in vitro*

**DOI:** 10.1039/d6pm00188b

**Published:** 2026-09-15

**Authors:** Apoorvi Chaudhri, Molli Garifo, Pranavi Thatavarthi, Torrick Fletcher, Jessica Larsen

**Affiliations:** a Department of Chemical and Biomolecular Engineering, Clemson University Clemson South Carolina USA larsenj@clemson.edu; b Department of Bioengineering, Clemson University Clemson South Carolina USA +617-632-5167 +617-632-4585

## Abstract

Glioblastoma represents a highly aggressive brain tumor with low survival and no response to chemotherapy and radiation therapy. Temozolomide, the current standard of care chemotherapy, improves patient survival by only about 6 months because of several resistance mechanisms, including unmethylated MGMT, which enables repair of chemotherapy-induced DNA damage. Thus, additional treatment strategies are necessary to investigate efficient responses towards glioblastoma. Doxorubicin (DOX) is a chemotherapeutic agent that is independent of MGMT methylation and instead works through inhibition of topoisomerase (TOPO) II, an enzyme necessary for DNA replication of the tumor. The inability of doxorubicin to cross the blood–brain barrier (BBB) precludes its use in glioblastoma. Polymersome nanoparticles have the potential to transport agents across the BBB. Here, we develop hyaluronic acid-*b*-polylactic acid (HA-PLA) polymeric nanoparticles called polymersomes, encapsulate them with DOX and investigate the ability of our system to induce apoptosis in a human glioblastoma cell line. HA-PLA polymersomes encapsulated with DOX at 6 µg mL^−1^ showed an encapsulation efficiency of 99 ± 1% and a PDI of 0.70 ± 0.02 with diameter of 122.1 ± 2.9 nm, with encapsulation efficiency decreasing and diameter increasing as additional DOX is added. Release studies showed an increase in DOX released from HA-PLA-based polymersomes due to acid-mediated hydrolysis of the PLA ester bonds. The HA-PLA polymersomes show specificity and receptor-mediated endocytosis towards CD44-positive U87 glioblastoma cells with ∼42% uptake due to the natural affinity of HA (hyaluronic acid) to CD44. This receptor-mediated and additional mode of endocytosis was greatly stunted at 4 °C. Jurkat cells that are CD44 negative did not show uptake. Our HLA-PLA-DOX system promotes apoptosis of glioblastoma through inhibition of topoisomerase (TOPO) II. Thus, our system could allow tumor specificity through HA–CD44 affinity and slow drug release through pH sensitivity of PLA in the acidic tumor microenvironment.

## Abstract 2

Hyaluronic acid-*b*-polylactic acid polymersomes encapsulate and deliver doxorubicin (DOX) to glioblastoma cells through the CD44 receptor. They degrade in an acidic tumor-mimetic environment to release DOX, causing cell death.

## Graphical abstract

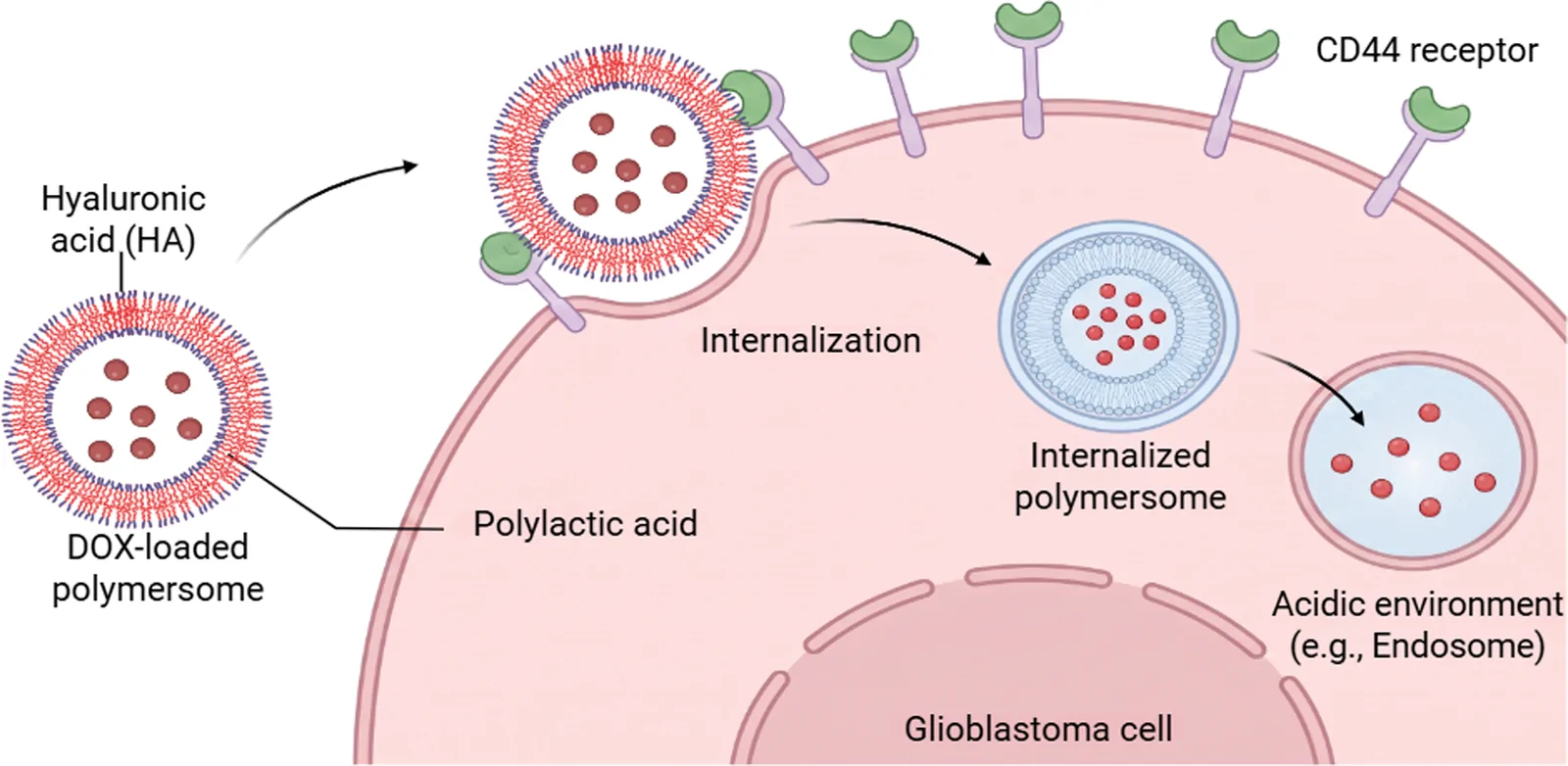

## Introduction

Glioblastoma is an aggressive tumor with a low survival rate of only 9 months.^1^ Temozolomide, the standard chemotherapy for glioblastoma, increases the survival rate to only 15 months.^2^ Resistance towards temozolomide includes unmethylation or overactivation of O6-methylguanine-methyltransferase (MGMT) that prevents DNA damage and cellular apoptosis.^3^ Doxorubicin is a chemotherapeutic agent that instead promotes tumor cell toxicity through the inhibition of topoisomerase II enzyme (TOPOII).^4^ Tumor cells are dependent on TOPOII for DNA replication and cell proliferation, a key event in cancer growth.^5^ Doxorubicin is a standard chemotherapy in multiple cancer types, including breast cancer, bladder cancer and non-small lung cancer.^6^ However, DOX results in serious side effects, including cardiotoxicity, myelosuppression, nausea, vomiting, alopecia, and mucositis.^7^ Thus, a need for targeted delivery of DOX with lower toxicity arises. Nanoparticles can act as delivery vehicles to effectively transport drugs across the BBB in multiple settings.^8,9^ Polymersomes are nano-vesicles that are formed *via* self-assembly of amphiphilic block copolymers.^10,11^ HA-PLA (hyaluronic acid-*b*-polylactic acid) derived polymersomes are formed from a block copolymer of hyaluronic acid and polylactic acid that self-assemble into vesicles based on their molecular weights.^12,13^ HA has a natural affinity to CD44 that facilitates nanoparticle internalization through receptor-mediated endocytosis in CD44+ tumor cells.^14^ Multiple tumor models have used HA nanoparticles to deliver agents in CD44-expressing tumor cells, including breast cancer, melanoma, and colorectal cancer.^15,16^

A major challenge to the use of doxorubicin in glioblastoma treatment is its penetration across the blood–brain barrier (BBB). It is hydrophilic, has a high molecular weight, and is a substrate for transporter proteins, resulting in low concentrations in the brain.^17^ This inability of doxorubicin to reach the glioblastoma tumor has prevented it from being an effective treatment strategy in this disease. PLA nanoparticles are engineered to release drugs in acidic tumor microenvironments *via* acid-mediated hydrolysis.^18^ Specifically, Deng *et al.* observed lower lung tumor metastasis in a 4T1 breast tumor xenograft model when treated with RGD-peptide conjugated HA-*b*-PLA-DOX. In mice, DOX had a higher blood circulation time when loaded in these nanoparticles.^19^ In addition, hyaluronic acid-*b*-polycaprolactone (HA-PCL polymersomes) loaded with DOX showed antitumor efficacy in 4T1 and MCF-7 metastatic breast cancer.^20^ Hyaluronic acid-*b*-poly (β-amino ester) (HA-PBAE polymersomes) exhibited pH-responsive release and strong tumor growth suppression in the 4T1 metastatic breast cancer model.^21^ Redox-responsive HA-SS- poly(lactic-co-glycolic) (PLGA) nanoparticles co-delivering DOX and cyclopamine reduced tumorsphere formation and improved survival in orthotopic breast cancer.^22^ Acetylated hyaluronic acid–poly(l-lactic acid) (AcHA-PLLA nanoparticles) loaded with DOX also demonstrated CD44-mediated uptake in HCT-116 colorectal cancer cells and reduced formation of the cardiotoxic metabolite doxorubicinol.^23^ Despite these advances, the application of HA-*b*-PLA polymersomes for DOX delivery specifically in glioblastoma remains limited. Thus, the efficacy of HA-PLA-DOX polymersomes should be investigated in glioblastoma. Advantages of HA-PLA polymersomes over free DOX include biodegradability, pH responsiveness, controlled release, targeted delivery and reduced toxicity.^13^ We hypothesize that DOX will be an effective therapy in glioblastoma when delivered through an HA-PLA based polymersome. Here, we generate HA-PLA polymersomes using solvent injection. Our HA-PLA polymersome with its hydrophobic PLA membrane encapsulates the hydrophilic DOX. We use dynamic light scattering on HA-PLA polymersomes loaded with or without DOX, and assess their diameter and surface charge. We perform release studies to examine the DOX release from HA-PLA in neutral and acidic pH to mimic both physiological and acidic tumor microenvironments. We load HA-PLA polymersomes with FITC-BSA and investigate their cellular uptake *via* the CD44 receptor in CD44+ U87 glioblastoma cells and CD44− Jurkat cells. Further, we evaluate the ability of HA-PLA-DOX on CD44+ U87 glioblastoma cells to reduce tumor cell proliferation using an MTS assay and perform analysis of the DOX-mediated TOPOII (topoisomerase II) inhibition in this cell type. Together, we investigate the ability of our HA-PLA-DOX system as a superior therapeutic strategy for efficient delivery of doxorubicin to CD44+ glioblastoma tumor cells.

## Experimental

### Materials

HA, with an average molecular weight of 7 kDa, was purchased from Creative PEGWorks (Durham, NC, USA). *N*-Hydroxysuccinimide (NHS) functionalized PLA (PLA-NHS) was purchased from PolySciTech, Akinalytics (West Lafayette, IN, USA) at an average molecular weight of 15 kDa. 1,4-Diaminobutane was purchased from Thermo Fisher Scientific (Waltham, MA, USA). Sodium cyanoborohydride (NaCNBH3, 99.2%) was purchased from Chem-Impex International (Wood Dale, IL, USA). *N*,*N*-Di-isopropyl ethylamine (DIPEA) was purchased from Thermo Fisher Scientific (Waltham, MA, USA). DOX was purchased from APExBIO (MW ∼0.5 kDa, Houston, TX, USA). Acetate buffer, dimethyl sulfoxide (DMSO), mannitol, sodium chloride, phosphate-buffered saline (PBS), 4-(2-hydroxyethyl) piperazine-1-ethane-sulfonic acid (HEPES) buffer were obtained from Sigma-Aldrich (St Louis, MO, USA).

U87-MG cells were purchased from ATCC (Manassas, VA, USA) to perform *in vitro* studies on malignant glioma cells. Standard cell media comprising of Eagle's Minimum Essential Medium (EMEM) with Earle's salts, non-essential amino acids, l-glutamine, sodium bicarbonate, fetal bovine serum (FBS), and 1× penicillin–streptomycin solution were purchased from Sigma-Aldrich (St Louis, MO, USA). Passages were performed using 0.25% trypsin (Corning, Inc., NY, USA). CellTriter 96 Aqueous One Solution Cell Proliferation (MTS) Assay was purchased from Promega (Madison, WI, USA). TOPO II Assay Kit was purchased from TopoGEN (Buena Vista, CO, USA). Quick-DNA Miniprep was purchased from Zymo Research (Tustin, CA, USA). Gel electrophoresis was performed using TAE buffer, synthesized following standard recipe using Tris base (≥99.8%) from Bio-Rad Laboratories (Hercules, CA, USA), glacial acetic acid (≥99%) from VWR (Radnor, PA, USA), and EDTA (≥99%) from Alfa Aesar (Ward Hill, MA, USA) and agarose from Thermo Fisher Scientific (Waltham, MA, USA). Ethidium bromide (EB) for gel staining was purchased from Thermo Fisher Scientific (Waltham, MA, USA). Albumin from bovine serum with fluorescein conjugate (FITC-BSA, MW ∼66 kDa) was purchased from Sigma-Aldrich (St Louis, MO, USA).

### HA-PLA synthesis

Hyaluronic acid-β-polylactic acid (HA-PLA) copolymer was synthesized in a two-step conjugation method as described in a previous study.^12^ The HA was selected to have a molecular weight of 7 kDa based on previous work performed in our laboratory. When enzyme-responsive polymersomes were made from HA 7 kDa and 15 kDa PLA, they exhibited a small nanoparticle diameter of 76.5 ± 7.7 nm, high loading capabilities using our osmotic pressure drive loading approach, and showed the greatest release rate variance between physiologic conditions and pathologic conditions.^12^

### Amination reaction of hyaluronic acid

A terminal reduction amination reaction was performed to functionalize HA for the conjugation step. In detail, 0.1 g of HA was dissolved in excess 0.1 M acetate buffer (pH 5.4) before adding 1 mL 1,4-diaminobutane dropwise under magnetic stirring. The amination reaction was ongoing for 24 hours at 50° C. NaCNBH_3_ (0.2 g) was added as a standard reducing agent with stirring at 50 °C. An additional 0.1 g of NaCNBH_3_ was added 24 hours after the first addition to ensure the reduction was complete. The mixture was dialyzed against deionized water for 72 hours to remove all excess unreacted 1,4-diaminobutane and NaCNBH_3_ using a 3.5 kDa molecular weight cut-off (MWCO) dialysis cassette (Spectrum Labs, CA, USA). The dialyzed mixture was then filtered using a 0.45 μm syringe filter (Millipore, MA, USA) before lyophilizing to obtain aminated HA in solid form.

### Conjugation of aminated hyaluronic acid and *N*-hydroxysuccinimide functionalized polylactic acid

The conjugation of PLA-NHS and aminated HA was performed using the NHS ester-amine coupling reaction.^12^ These procedures involved 0.3 g of PLA-NHS dissolved in 10 mL DMSO, and 0.2 g (stoichiometric excess) of animated HA added with 15 μL of DIPEA, which served as a catalyst for the coupling reaction. The reaction flask was housed in a sand bath and left to react for 48 hours, prior to heating at 50 °C inside a glove box to ensure the reaction occured under nitrogen. To remove all excess unreacted reactants, the reaction mixture was dialyzed against deionized water for 72 hours using a 10 kDa MWCO dialysis cassette (Spectrum Labs, CA, USA). The dialyzed mixture was then filtered using a 0.45 μm syringe filter before lyophilizing to obtain HA-PLA in solid form. Synthesis was confirmed using NMR, where HA-PLA polymer had similar peaks to free HA between 2.6 and 4.4 ppm and the addition of distinct peaks at 1.5 and 2.9 ppm, which correspond to PLA-NHS.^12^

### HA-PLA polymersome synthesis

The copolymer was used for polymersome synthesis *via* solvent injection. Briefly, HA-PLA was dissolved in DMSO at a concentration of 2.4 mg per 100 μL. An aqueous solution of 8 wt% mannitol in deionized (DI) water was used to enhance the polymersome stability during lyophilization. The polymer solution was injected into the aqueous solution using a syringe pump at a rate of 20 μL min^−1^ through a 20-gauge needle following previously established protocols from our laboratory.^24^ Synthesized polymersomes were filtered through a 0.45 μm syringe filter before characterization.

### HA-PLA polymersome characterization

Dynamic light scattering (DLS, Zetasizer Nano ZS90, Malvern Instruments, Malvern, UK) determined the particle size and ζ-potential of the polymersomes. One mL of the filtered polymersomes was used for size measurements at 37 °C. Sodium chloride was added to 1 mL of filtered polymersomes to obtain a concentration of 100 μM for ζ-potential measurements, as ions/counterions in solution are required to enable this measurement. The remaining polymersome solution was lyophilized for future characterization following previously established protocols.^25^ Polymersomes were prepared for transmission electron microscopy (TEM) at a concentration of 1 mg mL^−1^. Phosphotungstic acid at 1% was applied for negative staining. TEM images were taken on a Hitachi 7830 UHR transmission electron microscope (Tokyo, Japan) at 120 kV.

### Payload encapsulation in HA-PLA polymersome

Polymersomes were actively loaded with FITC-BSA or DOX at varying concentrations. Encapsulation efficiency (EE) and release profiles were calculated on a mass basis using UV-Vis analyses and appropriate internal calibration curves. Appropriate wavelength for UV-Vis analysis, a spectral scan was performed on DOX in DI water and pH 7.4 and 6.8 HEPES buffer at concentrations ranging from 0 to 24 μg mL^−1^ (Table S1). Lyophilized polymersomes were suspended into deioinized water at a concentration of 10 mg mL^−1^ containing 2 mg mL^−1^ FITC-BSA or known concentrations of DOX (3, 6, 12 or 24 μg mL^−1^, *C*_0_). 500 μL of sample was transferred into 100 kDa microcentrifuge dialysis tubes with 750 μL deionized water as the dialysate. The tubes were placed in a tube rack on a shaker plate for constant movement at room temperature. Every hour for four to six hours, the dialysate was analyzed using UV-Vis spectrophotometer to quantify the mass of DOX released (*C*_r_). The dialysate was replaced after every sample was collected to maintain a maximum concentration gradient. Any DOX not released through dialysis and not washed away from the surrounding fluid and the polymersome surface was assumed to be encapsulated. EE was calculated using the following equation:1
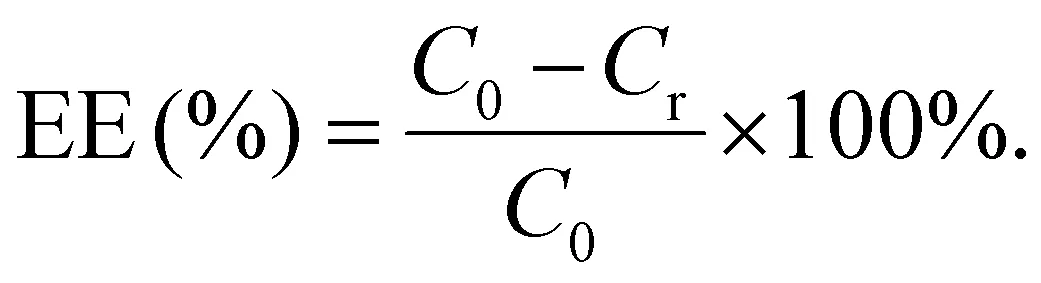


### Doxorubicin release from HA-PLA polymersomes

DOX-loaded polymersomes were concentrated using 100 kDa MWCO spin-down columns (Millipore, MA, USA). The concentrated, DOX-loaded polymersomes were resuspended in 100 μL pH 7.4 and pH 6.8 HEPES buffer to model both normal tissue and tumor microenvironments, respectively. The samples were transferred into 100 kDa mini dialysis tubes to allow transport of DOX but not the polymersomes, with 1.2 ml corresponding buffer as the dialysate. The tubes were placed in a tube rack on a shaker plate in an incubator at 37 °C to simulate physiological conditions. The dialysate was sampled over a minimum of 60 hours and analyzed using UV-Vis spectroscopy to quantify the mass of DOX released. Calibration curves are used to quantify the mass and percentage of DOX released.

### Cells and cell culture

U87-MG cells were maintained in EMEM with 10% heat inactivated FBS, 1% glutamax, and 1% pen-strep in 5% CO_2_. U87 cells were obtained from ATCC. Jurkat-NFAT cells were obtained from BPS technologies and maintained in BPS5 media; RPMI 1640 (Roswell Park Memorial Institute) with 10% heat inactivated FBS, 1% glutamax, 1% pen-strep, 1% HEPES, 1% pyruvate and G418 (500 µg ml^−1^) in 5% CO_2_. For polymersome uptake assays, Jurkat NFAT cells were plated in BPS4 media; RPMI 1640 (Roswell Park Memorial Institute) with 10% heat inactivated FCS, 1% glutamax, 1% pen-strep, 1% HEPES, 1% pyruvate in 5% CO_2_. These cell lines tested negative for mycoplasma. The U87 and Jurkat NFAT cell lines were assayed with anti-human CD44 antibody (Biolegend clone 1M7) using flow cytometry. The corresponding isotype was used as a control.

### HA-PLA polymersome cellular uptake

Polymersomes were loaded with FITC-BSA (MW ∼66 kDa, Sigma Aldrich) to study uptake behavior, as DOX would kill the cells. U87-MG cells or Jurkat-NFAT were seeded in a 96-well plate at 75 000 cells per well. After 24 hours, media was replaced and the wells were treated with the following groups: media (control), free FITC-BSA, or HA-PLA polymersome (2.5 mg), loaded at FITC-BSA concentration of 2 μg mg^−1^ polymersome. For Jurkat cells, the wells were pre-treated with poly-lysine to promote adherence. Treated cells were incubated for 4 hours at 37 °C or 4 °C. Upon incubation, cells were harvested and acquired on a Cytek Aurora (CA, USA) flow cytometer to quantify the uptake of HA-PLA polymersomes. Data was analyzed using FlowJo software version 10. Confocal microscopy was also used to visualize cellular uptake. Briefly, U87 or Jurkat cells were treated with conditions as described above for 4 hours at either temperature. After incubation, cells were fixed with ice-cold 4% PFA for 10 min on ice. On fixation, cells were washed with PBS, chambers were removed, and Vectashield antifade mounting medium with DAPI (Vector Laboratories H-1200-10) was added to the slides. The slides were sealed and incubated at least overnight at 4 °C before microscopy. Images were acquired using Leica Stellaris 5 confocal microscope. For competitive uptake experiments, varying amounts of HA-PLA-FITC-BSA and 10 mg mL^−1^ free HA were added during a 4-hour incubation at 37 °C. Uptake was analyzed by flow cytometry, with gates set to those of untreated controls.

### Calculation of LC-50 for free DOX

A dose escalation study was performed to determine which DOX concentrations should be encapsulated to achieve effective chemotherapeutic activity. U87-MG cells were seeded in a 96-well plate and allowed to sit overnight. Following, cells were treated with free DOX added to fresh growth media at final DOX concentrations 0–24 μg mL^−1^. At days 1–5 post-incubation, cell death was evaluated using the MTS proliferation assay. The MTS reagent was added to each well at 10% working volume and incubated for 4 hours. The absorbance was measured at 490 nm using a spectrophotometer (UV-Vis, BioTek Synergy H1, Aligent Technologies; CA, USA). LC50 values were calculated by fitting to a curve in GraphPad Prism.

### MTS cell viability assay

Viability studies were performed on empty and loaded polymersomes. U87-MG cells were seeded in a 96-well plate at 10 000 cells per well. When cells reached 70% confluency, cells were treated with 200 μL fresh media, 6 μg mL^−1^ and 24 μg mL^−1^ free DOX, and 6 and 24 μg mL^−1^ DOX-loaded polymersome. The viability studies were performed using an MTS proliferation assay 4 and 5 days after dosing as described in Ma *et al*.^26^

### Topoisomerase II assay

U87-MG cells were dosed with polymersomes in viability studies and collected after five days of incubation. DNA was extracted using the Zymo Quick-DNA Miniprep Kit. The TOPO II assay kit (TopoGEN Kit, TG1001) was used to detect the topoisomerase enzyme activity. The assay was performed based on the manufacturer's instructions. Briefly, the enzymatic reaction mixture contained 4 μL of 5× Complete Reaction Buffer, 200 ng kDNA substrate, 2 IU of TOPO II and 80–120 ng DNA extract from the treated cells. The mixture was incubated at 37 C for 30 min followed by addition of 5× Stop Buffer. The reaction products were run in a 1% agarose gel.

### Statistics

Results were graphed as mean with SEM (standard error of mean). The statistical analysis was performed using GraphPad Prism version 10 using 2 Way ANOVA and Tukey correction for multiple comparisons.

## Results

### HA-PLA polymersomes can be synthesized with uniform diameters

**Fig. 1:**
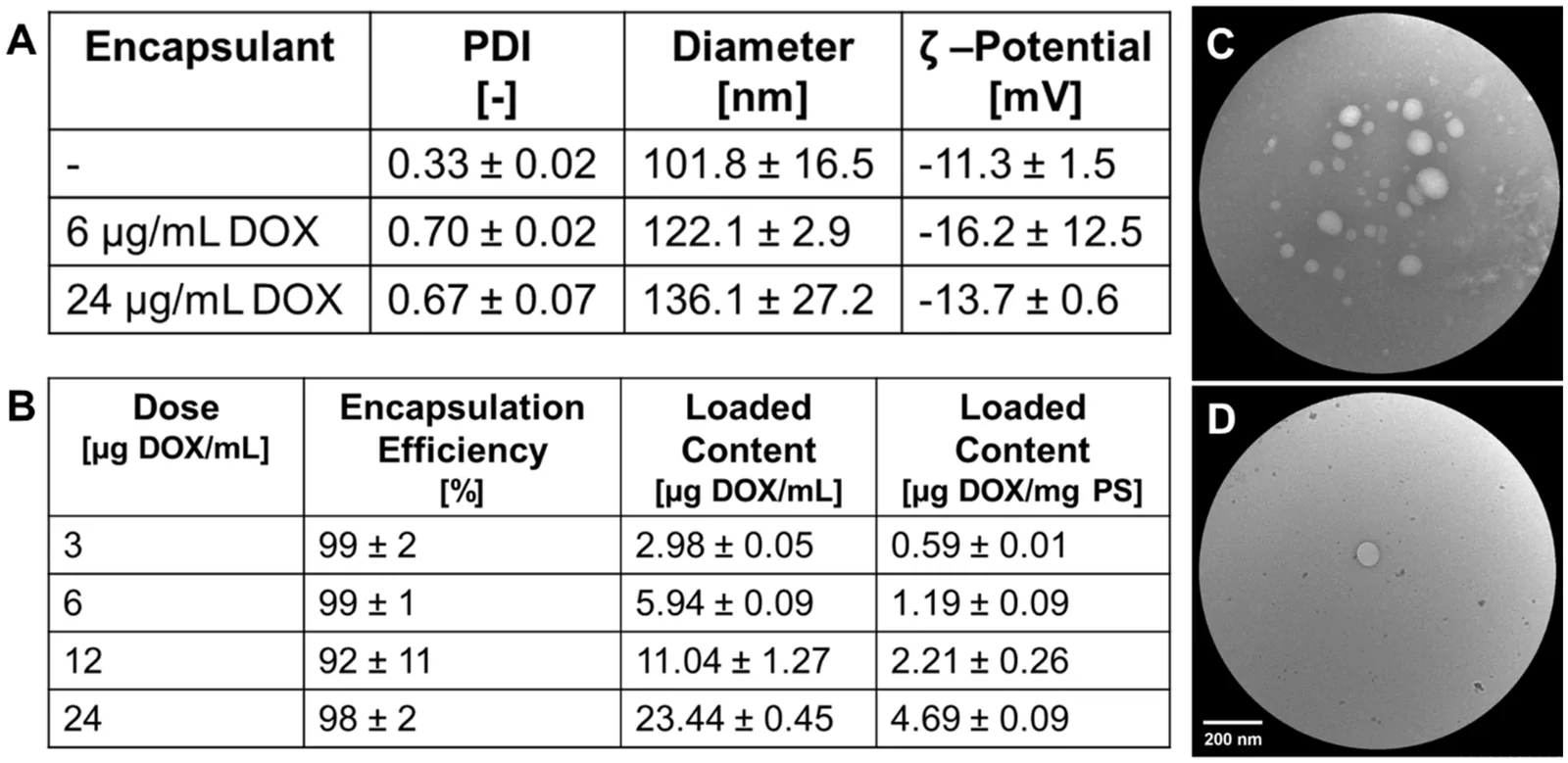
(A) Polymersome characterization *via* dynamic light scattering with and without DOX encapsulation. (B) Characterization of the encapsulation of DOX at various concentrations. (C) Representative TEM image of empty HAPLA polymersomes. (D) Representative TEM image of DOX-loaded HA-PLA polymersomes (6 µg mL^−1^).

We synthesized HA-PLA polymersomes using a solvent injection method as previously described in ref. 12. The polymersomes were characterized by dynamic light scattering and transmission electron microscopy (Fig. 1A, C and D). Empty HA-PLA polymersomes without DOX were included as a control. Empty HA-PLA polymersomes form at diameters around 100 nm with low polydispersity indices (PDI), while the addition of DOX appears to increase the polymersome diameter and the PDI. This increase in PDI is likely due to the innate fluorescence and colorimetric nature of DOX.^27^ DLS utilizes the material's refractive index to determine size and PDI; DOX naturally fluoresces red, rendering the solution colorimetric, which can be disruptive to sample measurement even when accounting for the changing solution properties in the software.^28^ Notably, TEM images identified relatively monodisperse particle diameters with DOX loading (Fig. 1D and Fig. S1). Overall, polymersomes, regardless of DOX loading, presented with an average diameter between 100–150 nm and a negative surface charge, suggesting effective uptake in the cells. A slightly negative surface charge can facilitate cellular uptake,^29^ specifically in malignant cell lines.^30^

### Doxorubicin can be encapsulated at high efficiencies and with high loading content into HA-PLA polymersomes

HA-PLA polymersomes were actively loaded with varying concentrations of DOX to ensure a therapeutically significant amount could be encapsulated and a maximum encapsulation was reached. 3, 6, 12, and 24 μg of DOX were added into a 1 mL sample containing 5 mg of lyophilized polymersomes to determine EE. All formulations achieved over 98% EE on average, aside from 12 μg, which had a very large deviation across replicates (Fig. 1B); however, all EE values were statistically similar to one another. The high EE is attributed to the addition of mannitol in the solvent during synthesis, as it acts as an osmotic agent.^31^ The solvent injection method produced a range in polymersome sizes (Fig. 1A), highlighted by the increasing standard deviation in diameters. This deviation in size could explain the slight deviation in EE. Loaded content was also calculated on a per mL and per mg polymersome basis, which provided useful information for designing *in vitro* and *in vivo* studies, as it allows for equal DOX dosing across various formulations. As expected, based on consistent EE, the loaded content increased proportionally with the amount of DOX initially loaded.

### Doxorubicin release is driven by diffusion and polymersome degradation

**Fig. 2:**
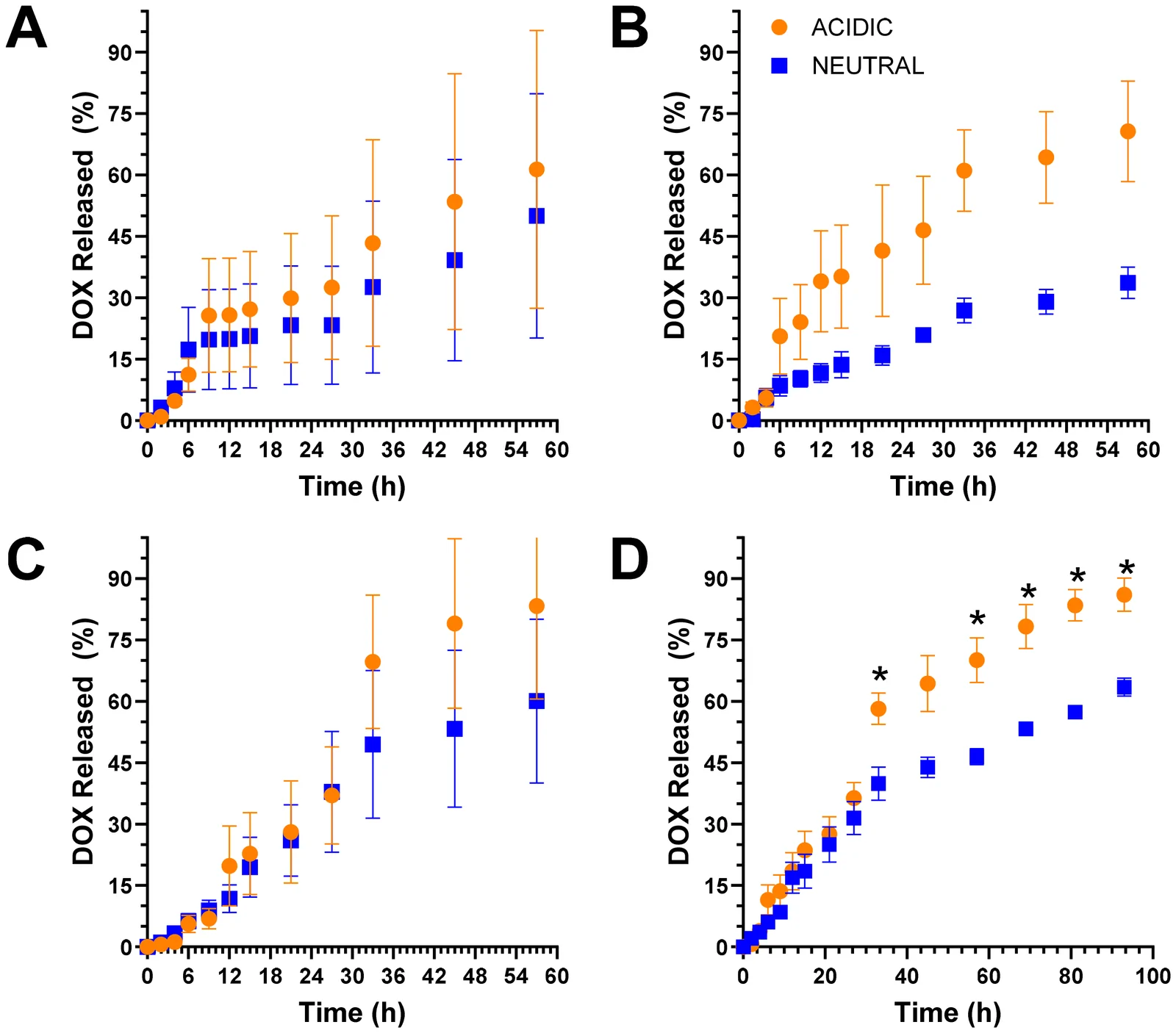
Release of DOX from HA-PLA polymersomes after various starting concentrations of DOX. (A) 3 µg mL^−1^, (B) 6 µg mL^−1^, (C) 12 µg mL^−1^, and (D) 24 µg mL^−1^.

Loaded HA-PLA polymersomes were incubated in physiologic and tumorigenic mimetic buffers. The acidic condition was 0.1 M HEPES Buffer at pH 6.85 to best represent the GBM tumor microenvironment,^32,33^ whereas the neutral condition was 0.1 M HEPES Buffer at pH 7.4 to represent the healthy brain environment. The study was performed for at least 60 hours. Fig. 2 illustrates the release profile for both acidic and neutral conditions. The lower concentrations of 3 (Fig. 2A), 6 (Fig. 2B), and 12 (Fig. 2C) μg DOX per mL resulted in little to no statistical significance in DOX released from HA-PLA polymersomes regardless of pH, likely due to the low overall DOX-loaded content; however, at 24 μg DOX per mL, statistically different release profiles were present between the buffer conditions after 36 hours (Fig. 2D). The low overall loaded content of DOX when only 3 µg mL^−1^ is added and the dramatic deviations observed in the loaded content of DOX when 12 µg mL^−1^ is added (Fig. 1B) are likely contributing to the large error bars observed in these release studies. For these reasons, further analysis included only HA-PLA polymersomes encapsulated with either 6 or 24 µg mL^−1^.

The lack of burst release at early time points suggests that DOX is encapsulated within the polymersome, as described by Wang *et al*.^34^ In the first few hours of release, regardless of the amount of DOX loaded, the release profile was similar across pH values, suggesting that early DOX release from polymersomes was diffusion-driven and required DOX to transport through the PLA membrane. This prolonged release of DOX is expected; Oz *et al.* observed a similar profile from PLA-based polymersomes in pH 7.4 PBS buffer, with a maximum cumulative DOX release of 70%.^35^ Multiple studies on the DOX release profile from PLA-based nanoparticles indicate an increase in release rate in more acidic environments, resulting from PLA degradation *via* acidic hydrolysis of ester bonds,^36–38^ which was also observed in our system.

**Fig. 3:**
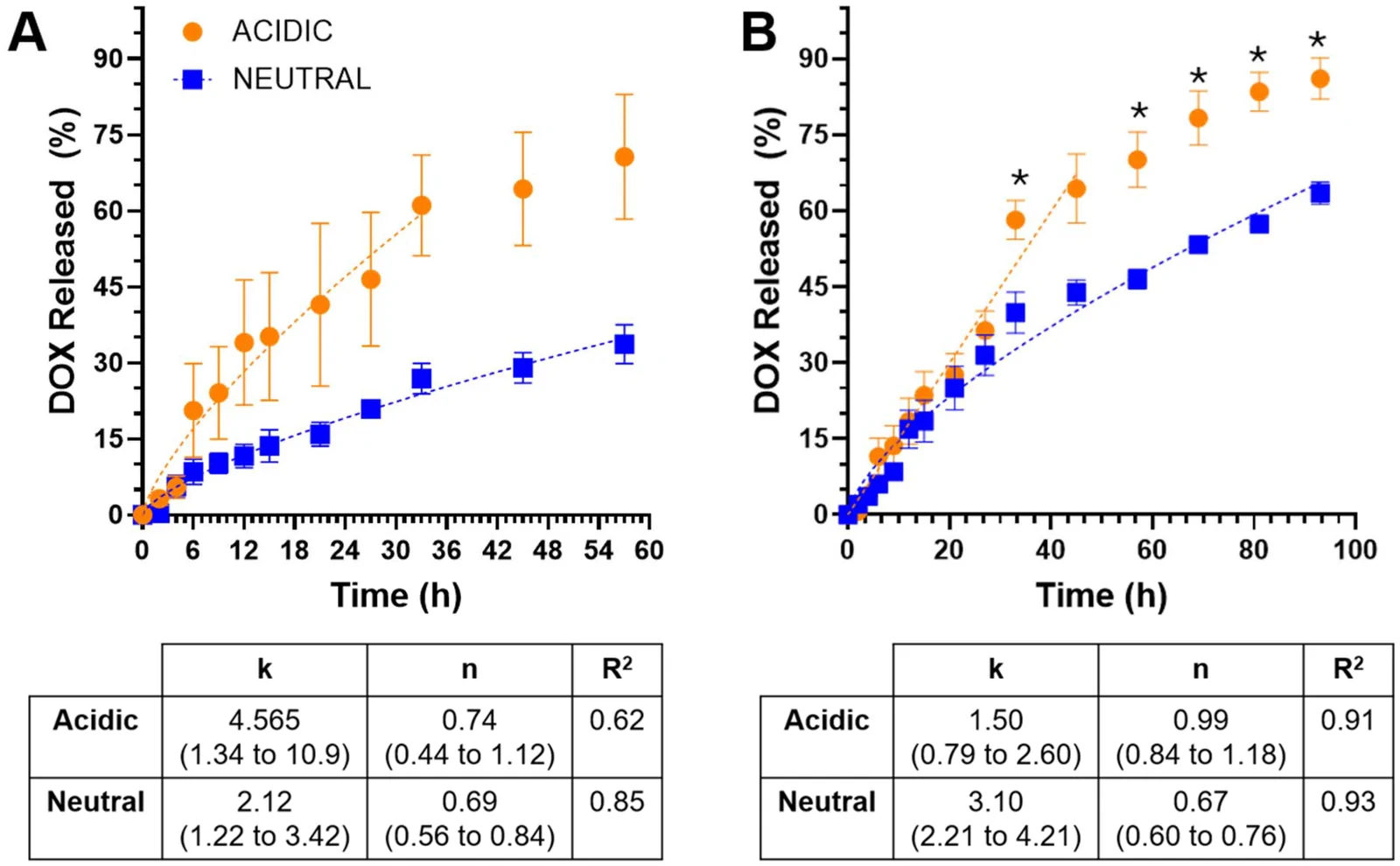
Release of DOX from HA-PLA polymersomes loaded with (A) 6 µg mL^−1^ or (B) 24 µg mL^−1^ is fit to the Korsemeyer–Peppas equation to highlight differences in release rates and release orders observed in each formulation.

The data was then fit up to 60% cumulative release to the Korsmeyer–Peppas model (eqn (2)) to establish the exponential relationship between release and time.^39^2
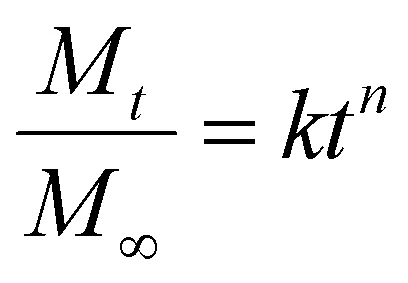
*M*_*t*_ represents the cumulative drug released at time *t*, *M*_∞_ represents the cumulative amount of drug released at infinite time, *k* is the release rate, and *n* represents the release exponent relating to the drug release mechanism. When *n* = 0.5, the release kinetics are described as Fickian drug diffusion.^40,41^ When 0.5 < *n* < 1, the model assumes non-Fickian release, and release can be driven by other properties like polymer relaxation/degradation. For our system, we expect to see 0.5 < *n* < 1 as initial drug release should resemble Fickian diffusion through the HA-PLA membrane, with a shift to polymersome degradation-drive release over time. As a result, *n* should be representative of non-Fickian diffusion overall, and *k* should be larger in acidic conditions as compared to neutral conditions to represent an increased release rate. The 6 µg mL^−1^ DOX formulation shows large differences in the release rate between acidic and neutral environments (Fig. 3A), suggesting PLA hydrolysis-driven DOX release that is more rapid in the acidic environment. When fitting to 60% cumulative release, the overall release rates and order of release vary more significantly when HA-PLA polymersomes incorporate additional DOX. Release rates from the 24 µg mL^−1^ DOX HA-PLA formulation appear to be slower in an acidic environment, while the release order is much closer to 1 or zero-order. However, post-24 hour release rates begin to differ, although the acidic release curve can no longer be fit using eqn (2), with higher apparent release rates in an acidic environment. This suggests that the first 24 hours of DOX release are driven by diffusion, while after 24 hours PLA hydrolysis-driven release becomes the driver (Fig. 3B).

### HA-PLA polymersomes are internalized by CD44 receptor-mediated endocytosis on U87 glioblastoma cells

**Fig. 4:**
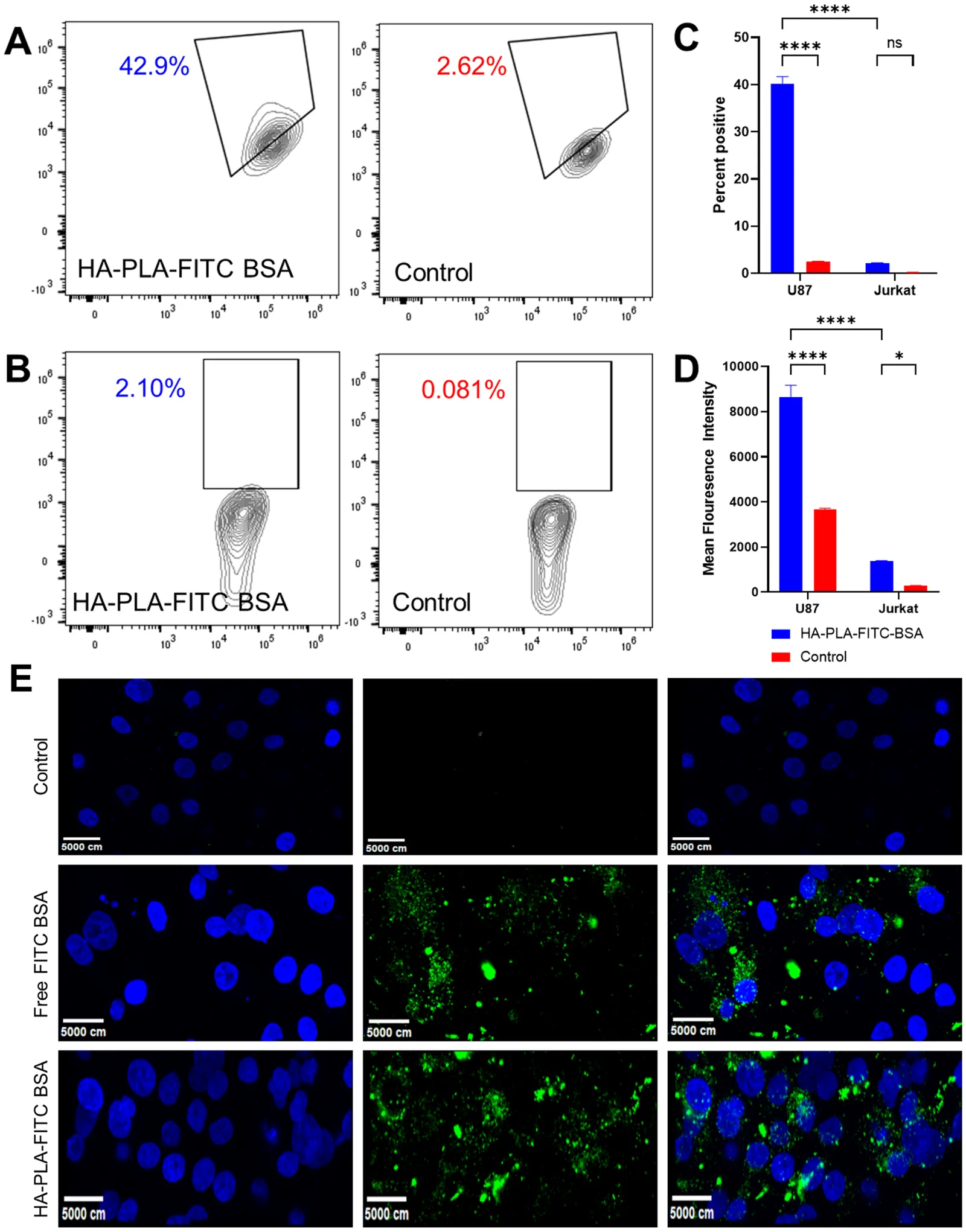
Example flow cytometry plots from HA-PLA-FITC BSA incubation with (A) U87 cells and (B) Jurkat cells at 37 °C. Flow cytometry data was analyzed over triplicates to calculate (C) the percent of cells positive for FITC-BSA and (D) the mean fluorescence intensity. (E) Confocal imaging confirms HA-PLA-FITC-BSA is taken up by U87 cells.* *p* < 0.05, ** *p* < 0.01, *** *p* < 0.001, **** *p* < 0.0001.

After confirming that U87 cells present with the CD44 receptor, while Jurkat cells do not (Fig. S2), we loaded HA-PLA polymersomes with FITC-BSA to determine the cellular uptake capability of HA-PLA polymersomes. It is important to note that uptake experiments using DOX-loaded polymersomes would not have been effective, as DOX can kill the cells. Similarly, 10 µL of 2 mg mL^−1^ FITC-BSA was added to 10 mg lyophilized polymersomes and the EE was determined to be 83.9 ± 6.7% with a loaded content of 1.68 ± 0.13 µg mg^−1^ HA-PLA, which is similar to our previous findings.^12^ To determine cellular uptake, U87 cells were treated with 2.5 mg HA-PLA polymersomes loaded with FITC-BSA and incubated at 37 °C for 4 hours. Polymersome uptake was examined by flow cytometry. HA-PLA-FITC-BSA showed approximately 41% cellular uptake, indicating successful internalization (Fig. 4A and C) with a high mean fluorescence intensity (MFI) (Fig. 4D). Confocal microscopy also showed internalization with HA-PLA polymersomes loaded with FITC-BSA and free-FITC-BSA, while no treatment did not show any green fluorescence, as expected (Fig. 4E). HA-PLA-FITC-BSA uptake in CD44-expressing U87 cells was compared with that in Jurkat cells lacking CD44 expression^42^ under the same temperature conditions and doses. Notably, uptake was minimal, with only around 2% of cells positive for polymersomes (Fig. 4B and C) and extremely low MFI (Fig. 4D).

**Fig. 5:**
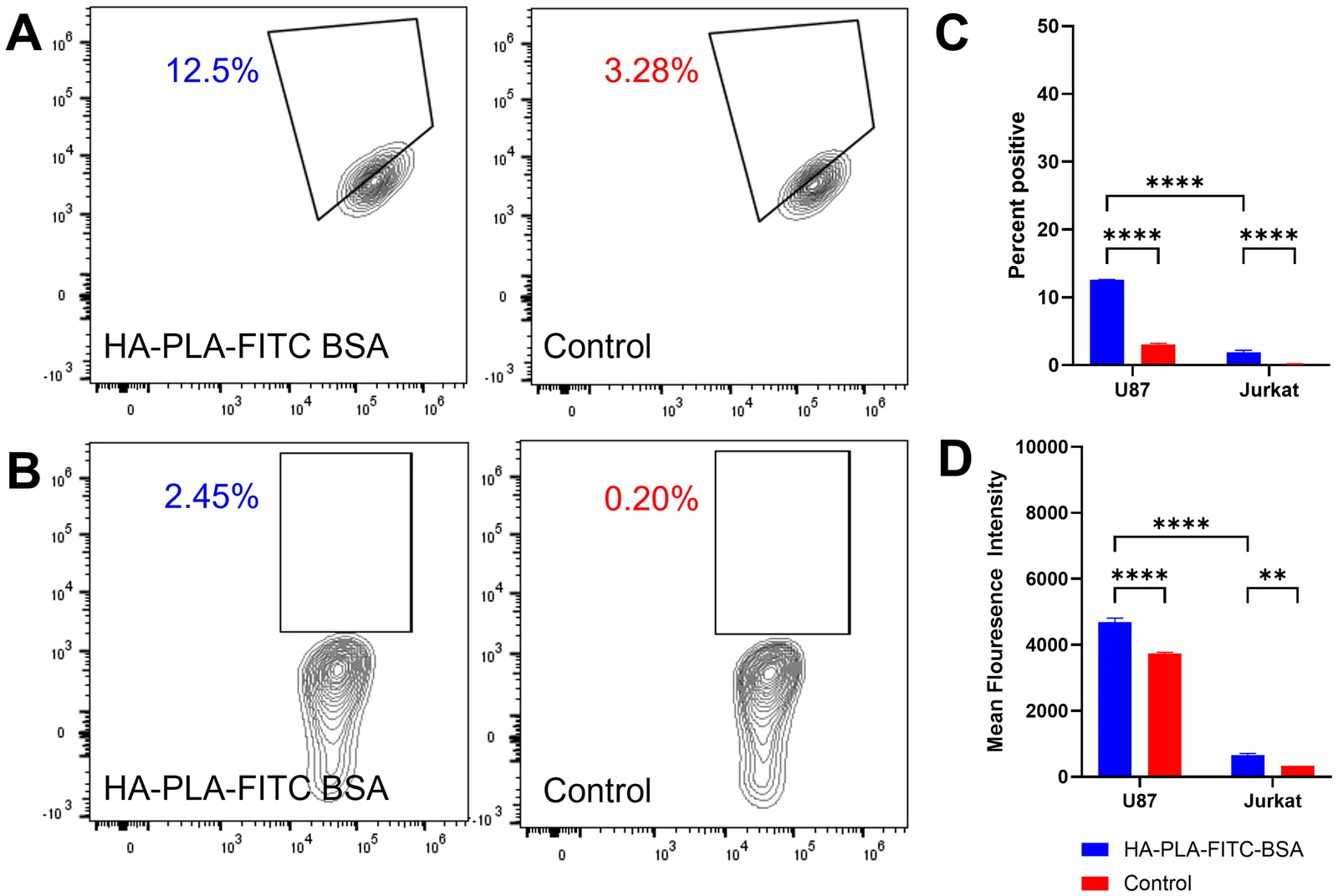
Example flow cytometry plots from HA-PLA-FITC BSA incubation with (A) U87 cells and (B) Jurkat cells at 4 °C. Flow cytometry data was analyzed over triplicates to calculate (C) the percent of cells positive for FITC-BSA and (D) the mean fluorescence intensity. * *p* < 0.05, ** *p* < 0.01, *** *p* < 0.001, **** *p* < 0.0001.

**Fig. 6:**
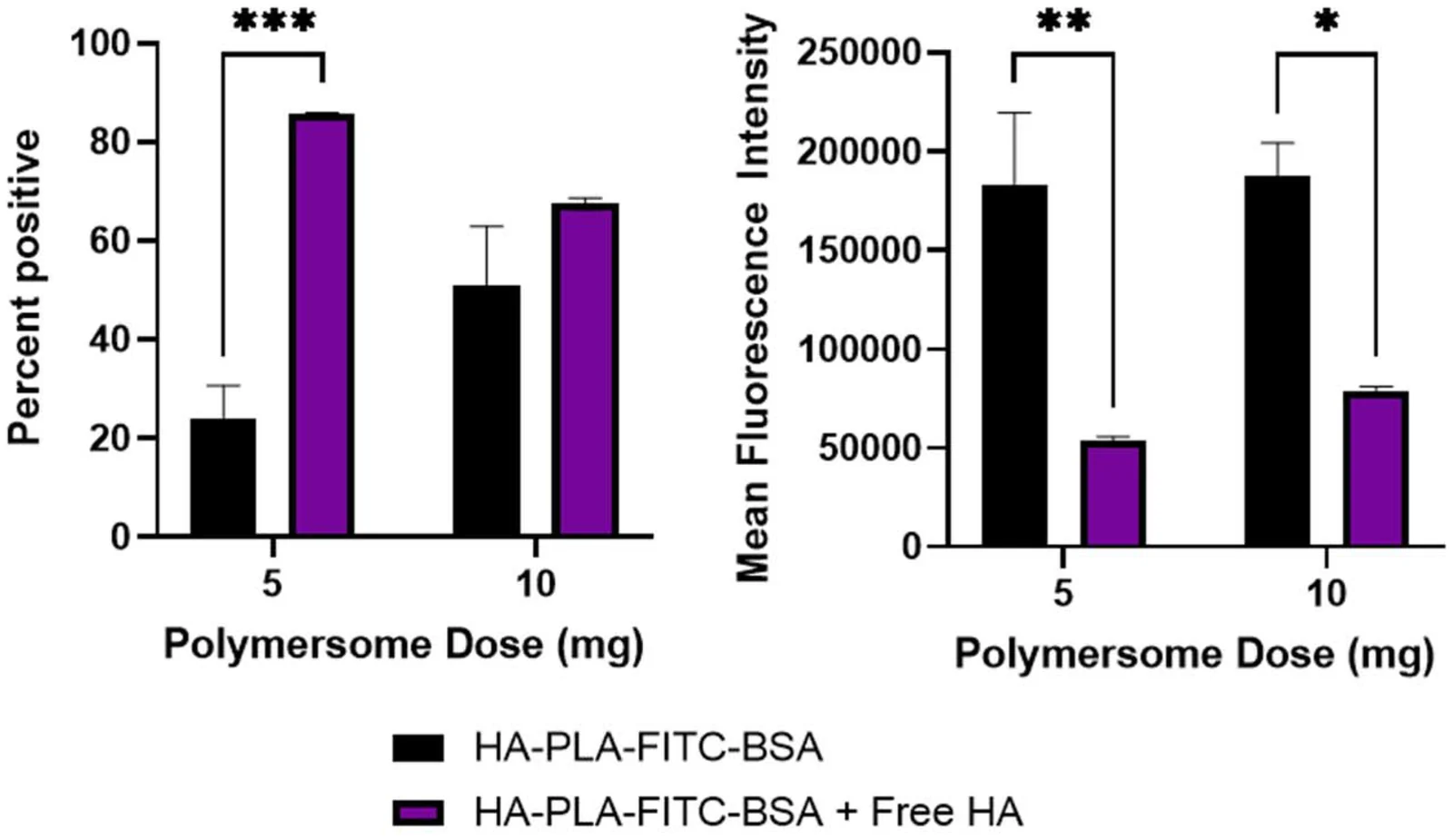
Flow cytometry data from competitive uptake experiments, where the addition of 10 mg mL^−1^ free HA (7 kDa) increased the number of positive cells (left) but dramatically decreased the overall mean fluorescence intensity (right).

Interestingly, treatment at 4 °C greatly reduced the uptake capacity of HA-PLA-FITC-BSA polymersomes in U87 cells from approximately 41% to approximately 12% (Fig. 5A and C) and around half the MFI (Fig. 5D). Changes in uptake between 37 °C and 4 °C were not as apparent with Jurkat cells with similar positive cells and MFI (Fig. 5B, C and D). Simultaneously, competitive uptake experiments changed the HA-PLA-FITC-BSA uptake behavior. While the average uptake of the polymersome is between 60–80% when in the competitive environment, the MFI was dramatically reduced, which indicated fewer polymersomes entered each cell (Fig. 6). Previous studies performed by Deng *et al.* utilized the addition of free HA within the cell culture media to evaluate CD44–HA relationship for cellular uptake. The addition of free HA provided a competitive environment for the HA-PLA polymersomes to bind to CD44 receptors on the surface of 4T1 cells, a metastatic breast cancer cellular model.^19^ The drastic reduction in MFI was similar to what was observed in this study, where a 14% reduction in MFI was observed after a one-hour incubation. We hypothesize the difference between our findings is a result of the significant increase in CD44 expression in GBM (98%) as compared to breast cancer cells (∼30%) and a longer incubation period.^43^ Together, our findings strongly suggest that the mechanism behind polymersome internalization was CD44 receptor-mediated endocytosis.

This uptake mechanism has been confirmed previously for lipid nanoparticles (LNPs) conjugated with HA, with breast cancer being the most widely studied.^44^ Specifically, Song *et al.* reported increased MFI for HA-functionalized LNPs loaded with DOX when administered to MCF-7 cells compared to HepG2 cells, due to increased CD44 expression in MCF-7 cells.^45^ Mahira *et al.* quantified CD44 expression levels in PC-3 and DU-15 cells (prostate cancer) before and after dosing with HA-coated LNPs, where a decrease in CD44 expression was observed post-treatment, suggesting the receptors were used for internalization.^46^ Shahriari, *et al.*, confirmed that HA-polycaprolactone based polymersomes were internalized in CD44 expressing 4T1 and MCF-7 cell lines at higher amounts prior to the addition of free HA, also suggesting this same mechanism.^47^

### Free doxorubicin performance

**Fig. 7:**
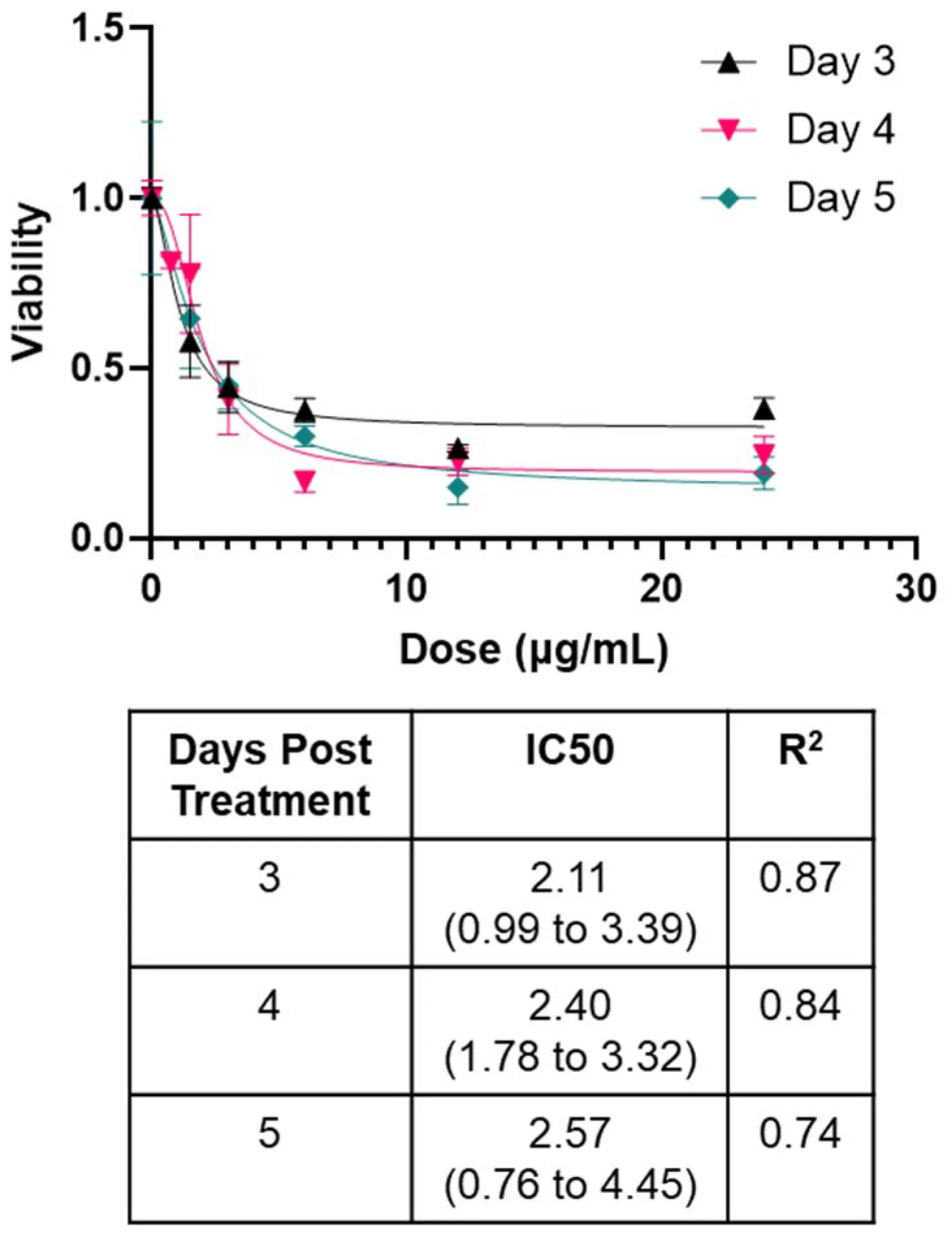
Viability of U87 cells over multiple doses of free DOX at 3, 4, and 5 days post-treatment is plotted to calculate the tabulated IC50 values.

To determine the concentration of DOX required to induce U87 apoptosis, cells were incubated with free DOX at increasing concentrations for 5 days. After 24 hours, the cells were 75% viable, indicating the requirement of a longer duration to induce apoptosis. It required at least 3 days post incubation to observe a viability that reduced to 50% (Fig. 3A). The viability data at 3,4, and 5 days post incubation of free DOX was fit to a curve to calculate the corresponding IC50 values, with values in the table reported as the 95% confidence interval (Fig. 7). The best fit IC50 value of free DOX over these three days averaged to be 2.36 ± 0.23 µg mL^−1^, indicating that our polymersome formulations with 6 and 24 µg DOX per mL, with loaded contents of 5.94 and 23.44 ± 0.45 µg DOX per mL respectively, should be sufficient to initiate apoptosis.

### HA-PLA-DOX polymersomes reduce viability of U87 cells

**Fig. 8:**
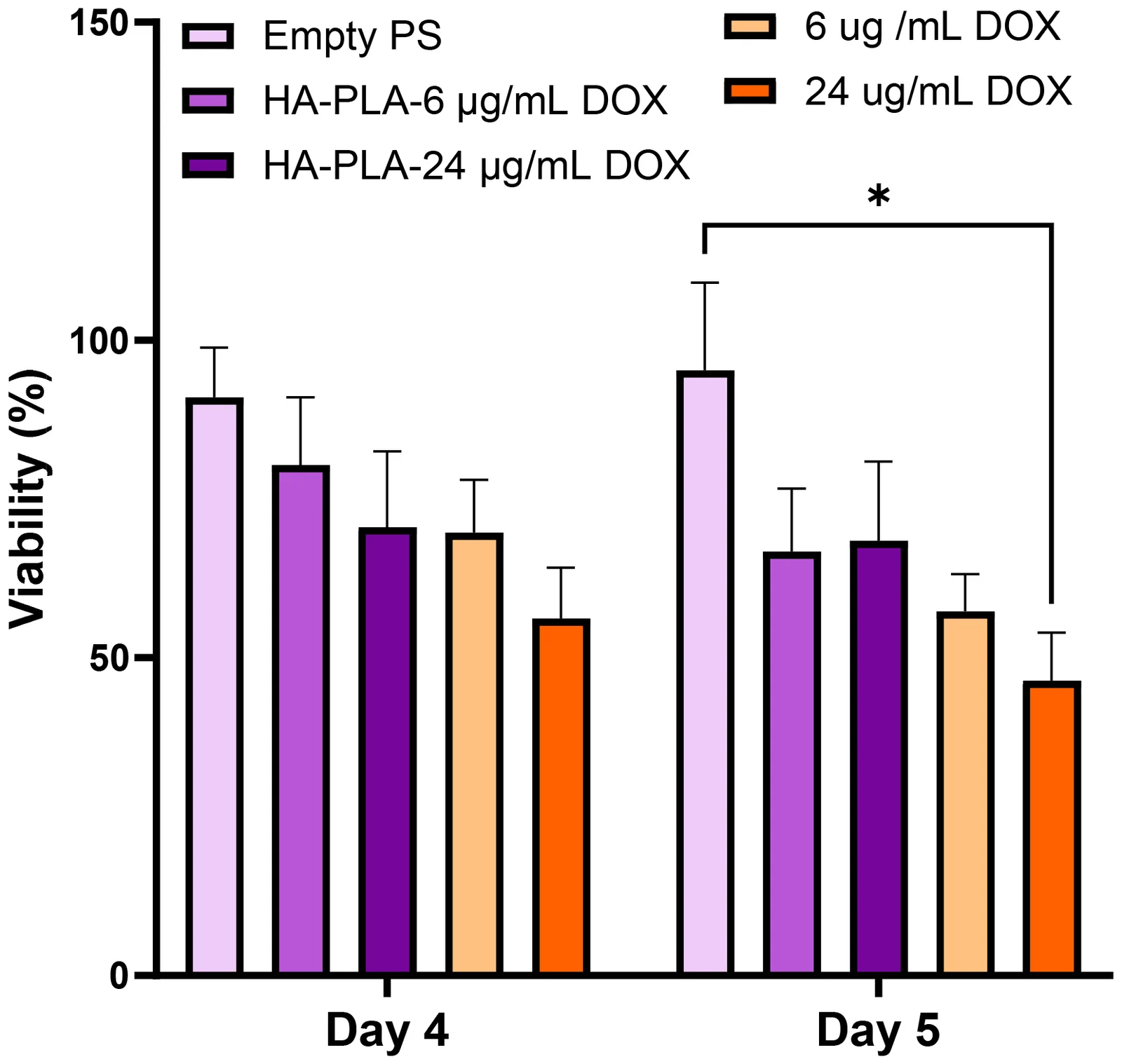
MTS viability assay was performed at 4 and 5 days post incubation with empty polymersomes (PS), various free DOX and HA-PLA DOX concentrations. *p < 0.05.

The safety profile of the HA-PLA polymersomes on U87 cells was evaluated using an MTS proliferation assay (Fig. 8). U87 cells were treated with 6 and 24 µg ml^−1^ of HA-PLA-DOX polymersome system for 4 and 5 days, with empty polymersome and free DOX as controls. High cell viability of 90% or greater was observed when treated with empty HA-PLA polymersomes as expected, indicating that the polymersomes itself without DOX did not pose any cellular toxicity.^12,48^ While free DOX was able to reach an IC50 at 5 days post incubation, neither HA-PLA-DOX formulation explored led to 50% viability or less. Notably, the viability of U87 cells was still decreased when DOX was delivered *via* polymersome, and the measured viability was not statistically different from free DOX at any time point. However, this increased viability could be due to the slower release of DOX from our polymersomes (Fig. 2 and 3); it is known that at least 3 days are needed to reach IC50 of free DOX (Fig. 7) and it is readily bioavailable in cells. An increased number of days may allow for IC50 values of HA-PLA polymersomes to be calculated. Similar behavior was observed when free DOX was compared to Doxil, an FDA-approved liposomal DOX formulation, with IC50 values of free DOX and Doxil calculated to be 0.25 µM and 0.7 µM, respectively.^49^

### HA-PLA-DOX induces inhibition of topoisomerase II in U87 cells

**Fig. 9:**
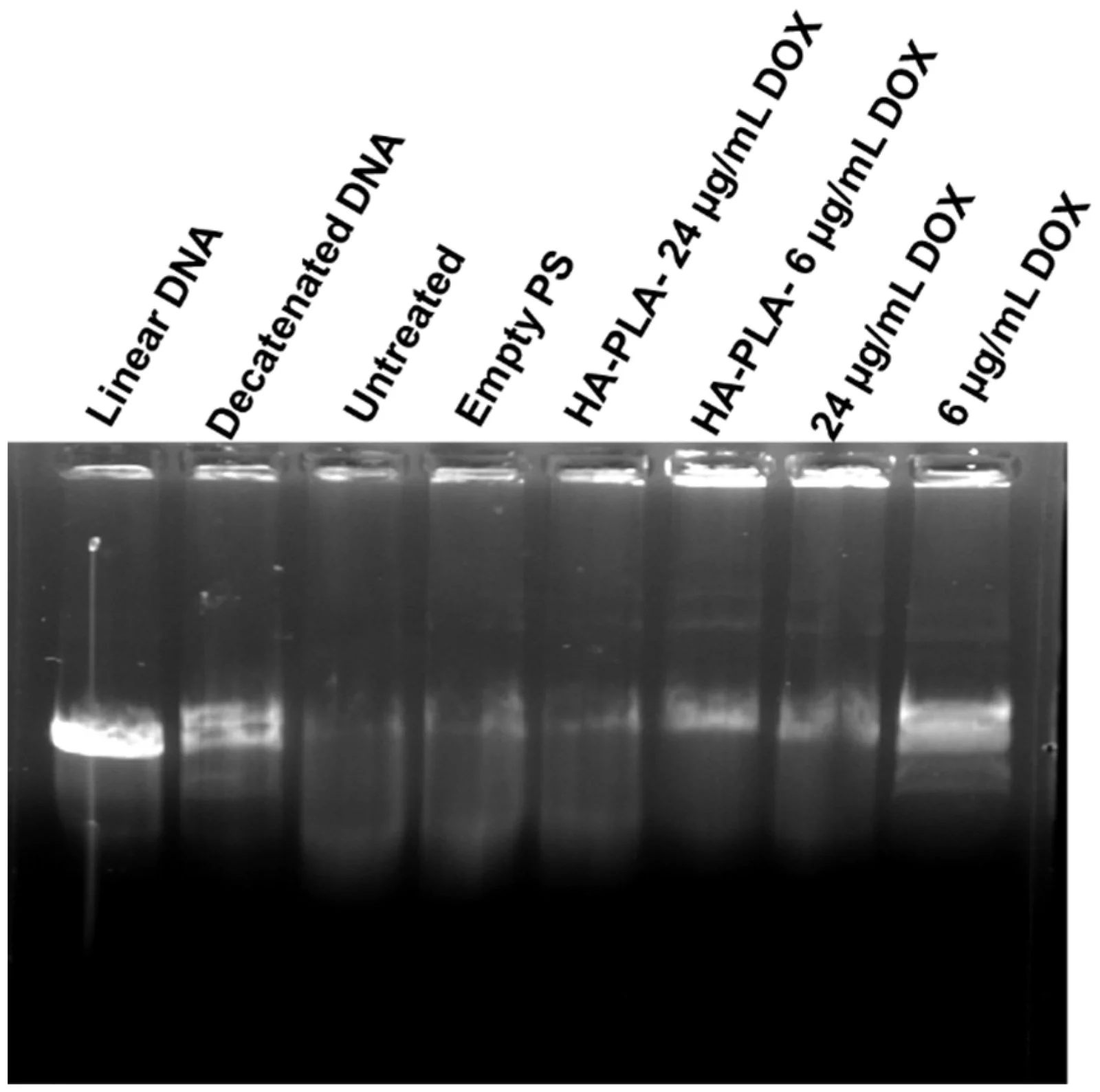
Ethidium bromide stained 1% agarose gel after electrophoresis to evaluate inhibition of TOPOII Activity. Lane 1: linear DNA marker (TOPOGen), Lane 2: decatenated DNA marker (TOPOGen). Lanes 3–8 contain DNA extracted from U87-MG cells 5 days after treatment. Lane 3: untreated, Lane 4: empty polymersome treated, Lane 5: 24 μg mL^−1^ DOX-loaded polymersomes, Lane 6: 6 μg mL^−1^ DOX-loaded polymersomes, Lane 7: 24 μg mL^−1^ free DOX, Lane 8: 6 μg mL^−1^ free DOX.

We treated U87 cells with HA-PLA-DOX system and extracted DNA followed by a test of topoisomerase activity using the TOPOII assay. After running gel electrophoresis (Fig. 9) on the reaction products with catenated and linear DNA as positive controls, the treated DNA extract of both free DOX and HA-PLA-DOX formulations produced significantly brighter bands retained in the well, indicating that inhibition of TOPOII occurred. This confirms that HA-PLA-DOX uses the same therapeutic mechanism as free DOX. While multiple bands may be seen further down the gel, this was representative of multiple types of decatenation products (nicked and intact circular); both indicated that TOPO II-induced decatenation. Literature reports the effect of DOX on TOPO II inhibition through TOPO II assays, evaluating varying concentrations of DOX on kDNA cleavage patterns.^50^ Complete inhibition would result in no products traveling down the length of the gel, indicating higher concentrations may be required for more significant results.^50–52^

## Conclusions

This study proposes a HA-PLA-DOX polymersome system for efficient delivery of chemotherapeutic agent doxorubicin in glioblastoma tumors. Polymersome-based delivery systems offer unique advantages for drug delivery, including specificity and biodegradability in human tissues. Several treatment strategies have not worked in glioblastoma due to constraints posed by the BBB. In summary, we have synthesized an HA-PLA copolymer that self-assembled into polymersomes through the solvent injection method. This method produced polymersomes with an average diameter of 101.8 ± 16.5 nm and ζ-potential of −11.3 ± 1.5 mV, characterized using TEM and DLS. These HA-PLA polymersomes achieved high encapsulation efficiencies of DOX above 98% when loading up to 24 µg DOX per mL. Our HA-PLA polymersomes were found to deliver DOX slowly over time, following a diffusion- and degradation-driven release model, with later time points favoring PLA hydrolysis in an environment mimicking the more acidic tumor microenvironment. Specifically, we find that HA-PLA polymersomes effectively infiltrate CD44-expressing glioblastoma cells *via* CD44 receptor-mediated endocytosis and effectively deliver DOX. We next confirmed that DOX-loaded polymersomes can reduce tumor cell proliferation in a human glioblastoma tumor cell line, U87, at 6 µg mL^−1^ and 24 µg mL^−1^ where they inhibit TOPOII. In this study, we observe efficient delivery of DOX through this nanoparticle system. Our work in an *in vitro* model of glioblastoma shows encouraging evidence of the potential for therapy. Subsequent work will focus on testing the efficacy of this system *in vivo* animal models of glioblastoma. Further, the ability of our HA-PLA polymersomes to cross the BBB will require ligand tagging to enable efficient trafficking and delivery at the tumor site. Overall, we expect this system to be efficient in glioblastoma through the CD44-mediated receptor endocytosis and acidic tumor microenvironment-mediated hydrolysis of the polymersome.

## Author contributions

Molli Garifo: writing – original draft, validation, investigation, formal analysis, data curation, conceptualization. Apoorvi Chaudhri: methodology, validation, formal analysis, investigation, writing – original draft, visualization. Pranavi Thatavarthi: validation, data curation. Torrick Fletcher Jr.: validation, data curation. Jessica Larsen: writing – review & editing, visualization, supervision, project administration, funding acquisition, conceptualization.

## Conflicts of interest

There are no conflicts to declare.

## Supplementary Material

PM-OLF-D6PM00188B-s001

## Data Availability

The data supporting this article have been included as part of the supplementary information (SI). Graphpad Prism files are provided for the uptake and release studies. The pdf provides the calibration curves used in release studies. additional TEM images. and validation of CD44 receptor expression on U87 and Jurkat cells. Supplementary information is available. See DOI: https://doi.org/10.1039/d6pm00188b.
